# Proceedings of Societies

**Published:** 1861-03

**Authors:** 


### PROCEEDINGS OF THE MEDICAL ASSOCIATION OF HANCOCK CO., ILL., JAN. 16, 1861.

The Association met. The President being absent, on motion Dr. H. Judd was called to the Chair. The proceedings of the last meeting were then read and approved; after which several very interesting cases of Diphtheria and Pneumonia were reported and discussed.

Drs. Pierson, Hall and Spitler were then appointed delegates to the American Medical Association, which will hold its next meeting in Chicago in June next. Drs. Hay, Boude and

Ellis were appointed alternates. Drs. Hollowbush, Hall and Powers were appointed delegates to the State Medical Convention, which meets in Jacksonville in June next. Alternates, Drs. Hoffman, Coolidge and Ferris.

Two members who were appointed on a Committee at the July meeting, to memorialize the Legislature to pass a Registration act, having failed in performing the duties of the Committee, Drs. Judd and Hall were appointed as substitutes, to act in conjunction with the Secretary on said Committee.

The President then appointed the following members to prepare and read essays at the next meeting of this Association, viz: Dr. Coolidge, on Hydrocephalus; Dr. Hall, on Diphtheria; Dr. Hollowbush, on Uterine Diseases; and Dr. Spitler, on Puerperal Convulsions.

On motion, it was ordered that the Secretary forward copies of the proceedings of this meeting to the offices of the *Chicago Medical Journal*, and the Hancock County papers for publication.

On motion, the meeting then adjourned to meet in Carthage on the third Wednesday in April next.

A. SPITLER, Secretary.

### ABSTRACT OF THE PROCEEDINGS OF THE WILL COUNTY MEDICAL SOCIETY,

AT ITS ANNUAL MEETING, FEB. 13, 1861.

The Society met, agreeable to order, at 2 o'clock P. M. of Wednesday, Feb. 13th, 1861, in the office of Dr. F. K. Bailey, Joliet. Dr. Bailey, President, in the Chair.

The following gentlemen were elected as officers for the ensuing year:

*President*—Dr. A. W. Heise.

*Vice President*—Dr. E. K. Willard.

*Secretary*—Dr. G. S. Thomas.

Drs. F. K. Bailey, P. B. McKay and M. J. Johnson were elected Censors.

The following are at present members of the Society:

Drs. W. Danforth, J. R. Casey, F. K. Bailey, A. W. Heise, G. W. Thomas, Joliet; E. K. Willard, P. B. McKay, S. M. Abbot, A. W. Bowen, Wilmington; O. Corben, Plainfield; Prof. A. L. McArthur, (Lind University) Joliet; Drs. J. F. Dagget, Lockport; H. Folke, Wilton; M. J. Johnson, E. H. Strong, Wilmington.

Dr. A. W. Heise read an interesting paper upon Placenta Prævia, which was listened to with much pleasure by all present.

Dr. P. B. McKay was appointed to read an essay before the Society at the next meeting.

On motion, adjourned to meet at 1 o'clock Wednesday P. M., March 13th, at the office of E. K. Willard in Wilmington.

A. W. HEISE, President.

G. S. THOMAS, Secretary.

### AMERICAN MEDICAL ASSOCIATION.

The fourteenth annual meeting of the American Medical Association will be held in Metropolitan Hall, city of Chicago; commencing on the *first* Tuesday in June next.

Each regularly organized Medical Society is entitled to send one delegate for every ten of its members; and each Medical College is entitled to two delegates. It is desired that the names of delegates should be forwarded to the undersigned, as soon after their appointment as practicable.

H. A. JOHNSON, *Assist. Secretary.*

Chicago, Feb. 1st, 1861.

